# The full mitochondrial genome sequence of the greater argonaut *Argonauta argo* (Cephalopoda, Argonautoidea) and its phylogenetic position in Octopodiformes

**DOI:** 10.1080/23802359.2021.1911710

**Published:** 2021-04-27

**Authors:** Kazuki Hirota, Masa-aki Yoshida, Takehiko Itoh, Atsushi Toyoda, Davin H. E. Setiamarga

**Affiliations:** a Department of Applied Chemistry and Biochemistry, National Institute of Technology (KOSEN), Wakayama College, Wakayama, Japan; b The University Museum, The University of Tokyo, Tokyo, Japan; c Oki Marine Biological Station, Shimane University, Oki Island, Japan; d School of Life Science and Technology, Tokyo Institute of Technology, Tokyo, Japan; e Comparative Genomics Laboratory, National Institute of Genetics, Mishima, Japan; f Advanced Genomics Center, National Institute of Genetics, Mishima, Japan

**Keywords:** Paper nautilus, shelled octopods, mitogenome, Oki Island, Sea of Japan

## Abstract

The greater argonaut *Argonauta argo* is a species of the paper nautilus (Argonautidae), which is a family in Octopoda. In this paper, we report its full mitogenome sequence, which was obtained from a specimen collected in the Japan Seas near Oki Island, Shimane Prefecture, in Japan. The sequence was determined using the NGS Illumina HiSeq platform. With its 37 genes, the mitogenome shows a typical metazoan and Octopoda genomic structure, and similar to the mitogenome of the previously reported congener, *A. hians*. To confirm *A. argo* phylogenetic position in Octopoda, we conducted maximum likelihood phylogenetic analysis, using a data set including publicly available 17 Octopodiformes, five Decapodiformes, three Nautiloids and two outgroup Conchiferans. The result confirmed the affinity of Argonautidae to *Tremoctopus*, and the sister group position of this clade against the rest of incirrate Octopods. The mitogenome and phylogeny of *A. argo* reported here will be useful for future studies involving this enigmatic species, including on the reacquisition of external calcified shell structures in mollusks.

The greater paper nautilus *Argonauta argo* is the largest species of the sole extant genus of the pelagic octopod Argonautidae (Young et al. 1998). Similar to its congener, *A. hians*, it is a cosmopolitan species inhabiting the tropical and subtropical open seas worldwide (Norman 2000). Also similar to all females of the genus, female *A. argo* produces a thin, brittle calcite egg cases formed by two specialized dorsal arms, which outer form resembles the shape of Nautiloids’ and Ammonoids’ shells (Scales 2015; Stevens et al. 2015). This ‘shell’ (egg case) is thought not to be a homologous structure to the calcified shells of Conchiferans and Cephalopods, but an evolutionary innovation (an apomorphy) of the genus (Naef 1923).

In this paper, we report the full mitochondrial genome (mitogenome) sequence of *A. argo*. A piece of the gonad (ovary) was collected from an individual female specimen and was stored frozen at −80 °C until DNA extraction. The individual was collected in 2018 from the Sea of Japan around Oki Island (36°17′20.6″N, 133°12′46.4″E) of Shimane Prefecture in Japan. The shell is registered as a collection of The University Museum, The University of Tokyo in Tokyo, Japan (Voucher No. RM33391). Genomic DNA was extracted from the ovary using the QIAGEN Genomic-tip kit. The DNA sample was then analyzed on a HiSeq Illumina Next Generation Sequencer. To reconstruct the full mitochondrial genome, we performed contig assembly (-n 200) with Platanus v1.2.4 (Kajitani et al. 2014) using the paired-end data. Contigs annotated as mitochondrial sequences were extracted by using the mitogenome data of a closely related species, *A*. *hians* (NC_036354), as the query for BLASTn homology search. After assembling the contigs, both ends of the resulting single contig were manually confirmed to overlap, and redundant parts were removed to complete the full circular mitogenome. Afterward, the full mitogenome sequence was annotated using the web server version of MITOS (Bernt et al. 2013).

*Argonauta argo*’s mitogenome sequence was 15,741 bases-long (registered to DDBJ; Accession Number LC596061). Its structure is identical to the previously reported mitogenome of *Argonauta hians* (Chiu et al. 2018) and to those of the order Octopoda in general (Cheng et al. 2013; Magallón-Gayón et al. 2020). Detailed genomic structure is as follows: (1) There are 13 protein-coding, two rRNA, and 22 tRNA genes; (2) One control region is located between the COX3 and tRNA-Glu (817 bp-long); (3) Seven of the 13 protein-coding genes are coded on the L chain (ATP6, ATP8, COII, COI, ND2, ND3, and COIII); (4) ND4L is located in tandem with ND4 on the H chain; (5) Both the SSU-rRNA and LSU-rRNA coding genes are located on the H chain; (6) eight tRNA genes (tRNA-Ser(agc), tRNA-Thr, tRNA-Ile, tRNA-Asn, tRNA-Arg, tRNA-Ala, tRNA-Lys, and tRNA-Asp) are located on the L chain. The total GC content of the mitogenome was 23.3%, similar to other members of Octopoda (Cheng et al. 2013; Magallón-Gayón et al. 2020). To check the phylogenetic position of *A. argo* among the octopods, we collected mitogenome sequences of one polyplacophoran and one gastropod as outgroups, three nautiloids, five decapodiforms, and 17 octopodiforms from GenBank, and included them in the data set for phylogenetic analyses. A maximum likelihood phylogenetic analysis (Yang 1994) using the GUI version of RAxML (Silvestro and Michalak 2012; Stamatakis 2014) was conducted. Detailed methods of the analysis are shown in the legend of Figure 1.

**Figure 1.**
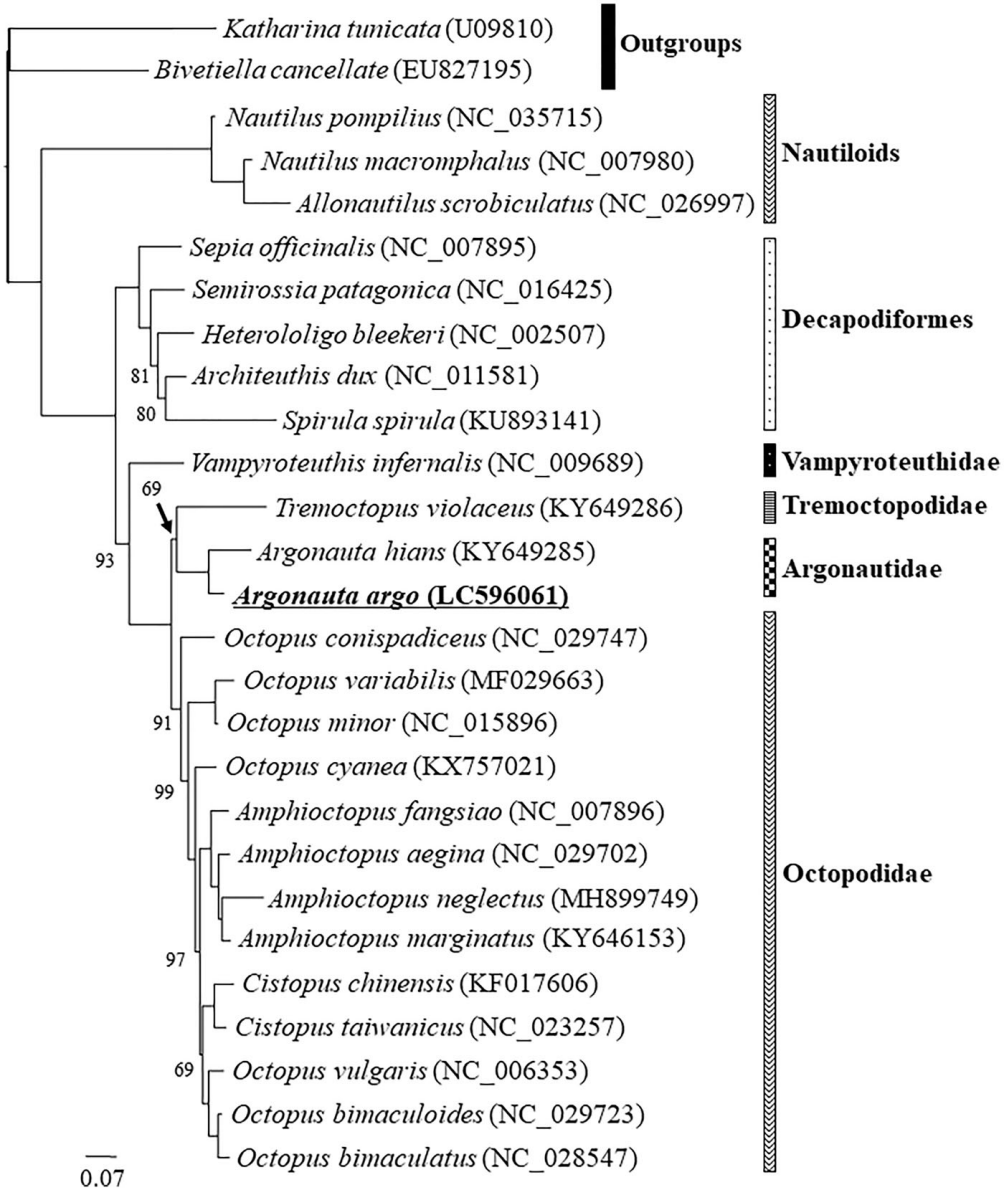
The maximum likelihood (ML) phylogenetic tree showing the position of *Argonauta argo*. The support values of nodes with 100% bootstrap supports are not shown. Phylogenetic analyses were conducted on a data matrix (10,428 positions) including all concatenated nucleotide sequences of the mitogenomes except the third codon positions of the protein-coding genes. Gene sequences were aligned individually using the online version of MAFFT under default settings (Katoh and Standley 2013). Aligned sequences were individually edited using the online version of GBlocks using the least stringent settings (Castresana 2000). A partitioned ML analysis (three partitions: protein-coding, rRNA, tRNA) were performed using the RAxML-GUI ver. 1-5b1 (Silvestro and Michalak 2012; Stamatakis 2014), with the GTR + Γ nucleotide substitution model (Yang 1994) 1000 rapid bootstrap replications.

The topology of the obtained ML tree is shown in Figure 1. The resulting phylogeny showed that *A. argo* formed a group to its congener, *A. hians*, forming a monophyletic Argonautidae, with the blanket octopus *Tremoctopus* as its sister group (superfamily Argonautoidea) (Figure 1) (Strugnell et al. 2006; Sanchez et al. 2018). Our phylogenetic tree also showed that Argonautoidea is sister to the rest of incirrate Octopoda (Uribe and Zardoya 2017; Chiu et al. 2018). We are confident that the result presented here will be useful for future molecular phylogenetics studies addressing taxonomic and systematic questions in Argonautidae, Argonautoidea, and Octopoda, beside for future phylogeography and population genetics studies of this species.

## Data Availability

The full mitochondrial genome sequence reported in this study is registered in and openly available from the National Center for Biotechnology Information (NCBI) Genbank database (Accession No.: LC596061; https://www.ncbi.nlm.nih.gov/nuccore/LC596061.1). The shell (eggcase) of the specimen was deposited at The University Museum of The University of Tokyo, Japan (Takenori Sasaki; sasaki@um.u-tokyo.ac.jp) under the voucher number UMUT-RM33391.
